# Molecular Diagnostic Assay for Rapid Detection of Flag Smut Fungus (*Urocystis agropyri*) in Wheat Plants and Field Soil

**DOI:** 10.3389/fpls.2020.01039

**Published:** 2020-07-10

**Authors:** Prem Lal Kashyap, Sudheer Kumar, Ravi Shekhar Kumar, Anju Sharma, Poonam Jasrotia, Devendra Pal Singh, Gyanendra Pratap Singh

**Affiliations:** ICAR—Indian Institute of Wheat and Barley Research, Karnal, India

**Keywords:** diagnosis, internal transcribed spacer, molecular detection, PCR, primer, sensitivity

## Abstract

Flag smut incited by *Urocystis agropyri* has the potential to cause substantial reduction in yield and quality of wheat production. An early and precise diagnosis is a key component in the successful management of flag smut of wheat. Therefore, a simple molecular assay for the rapid detection of *U. agropyri* was developed for the first time. To detect *U. agropyri*, species specific primers were developed by comparing the partial sequences of internal transcribed spacer (ITS) DNA region of *U. agropyri* with related and unrelated phytopathogenic fungi. The clear amplicons of 503 and 548 bp were obtained with the two sets of designed primers (UA-17F/UA-519R and UA-15F/UA-562R) from the genomic DNA of 50 geographic distinct isolates of *U. agropyri*. However, no amplicon was obtained from the DNA of other 21 related and unrelated phytopathogenic fungi which showed the specificity of the primers for the *U. agropyri*. PCR reaction was also set up to confirm the presence of *U. agropyri* spores in six different wheat varieties along with eleven distinct regional soil samples as template DNA. The presence of *U. agropyri* in all the soil samples collected from an infected field and plant tissue of diseased plants collected at two different stages (20 and 40 days post sowing) and the absence in the soils and plants of healthy plots indicated 100% reliability for detection of *U. agropyri*. This simple and rapid test can be employed for the detection of *U. agropyri* from enormous wheat and soil samples in very short time with less man power. Thus, the reported molecular assay is very specific for *U. agropyri* and requires less time and man power over conventional diagnosis which is often confused by coinciding morphological features of closely related fungal pathogens, and therefore, it can be used for quarantine surveillance of flag smut.

## Introduction

Globally, wheat (*Triticum aestivum* L.) is one of the most important staple food crops, but its production is adversely affected by numeral biotrophic fungi from sowing to harvesting. Many of these are also reported as important quarantine pathogens. Rossman et al. (2006) categorized *U. agropyri* as a ‘Threat to Major Crop Plants’ and advocated restriction on wheat and wheat straw imports in North America (Chalkley, 2020). Historically, it was first documented from South Australia and later on from Chile, China, Egypt, India, Japan, Mexico, Pakistan, South Africa, and USA (Toor et al., 2013; Savchenko et al., 2017). *U. agropyri* cause systemic infection and produce sori in the form of narrow stripes between the leaf veins at the fourth- to fifth-leaf stage and is the first authentic indication of *U. agropyri* infection in wheat (Mordue and Waller, 1981; Kruse et al., 2018). The infected plants produce malformed inflorescences and as a consequence are unable to produce seeds (Purdy, 1965). The seed and soil-borne nature of the disease leads to gradual built up of the inoculum in the soil (Ram and Singh, 2004), and teliospores can remain viable for 7–10 years in soil. Under congenial environmental conditions, the pathogen may cause complete yield loss (Hori, 1907; Zhao et al., 2019). Several reports indicated that because of the incessant nature of the pathogen and the cultivation of susceptible varieties, flag smut may become a serious threat for sustainable wheat cultivation (Shekhawat and Majumdar, 2013; Kumar et al., 2019). Up to 20% of crop loss was reported in the USA, Iran, Italy, and Egypt (Purdy, 1965). Yu et al. (1936) reported 90–94% infection in China. A yield loss of 5% was reported in India by Padwick (1948). Beniwal (1992) documented 23–65% yield losses from flag smut infection in nine commercial wheat cultivars. Moreover, reduction in tillering, plant height, ear head length, and 1,000 grain weight were recorded to the tune of 15–45%, 37–62%, 28–46%, and 19–37%, respectively. Thus, it becomes crucial for the timely management of this disease to harvest good wheat production because there is no suitable and effective control measure after seed sowing. The most effective management strategy for flag smut of wheat is seed treatment and a number of fungicides, *i.e.* copper carbonate (Fischer and Holton, 1957), quintozene (Yasu and Yoshino, 1963), benomyl, carboxin, and oxycarboxin (Metcalfe and Brown, 1969; Goel et al., 2001) were found effective. In addition, fenfuram, triadimefon, triadimenol, tebuconazole, and difenoconazole have been found effective in the control of *U. agropyri* (Goel and Jhooty, 1985; Tariq et al., 1992; Shekhawat et al., 2011; Singh and Singh, 2011; Shekhawat and Majumdar, 2013; Kumar et al., 2019). However, injudicious and long term usage of agrochemicals contaminates the ecosystem, threatens human and animal health, and leads to the emergence of fungicide resistance (Meena et al., 2020). Therefore, timely identification and detection of *U. agropyri* becomes imperative.

The spores of flag smut of wheat pathogen (*U. agropyri*) are similar to several other species of smuts and bunt fungi attacking wheat and triticoid grasses, which makes the identification of wheat flag smut spores very difficult on the basis of conventional morphological and culturing methods either in the soil or if the host is not accurately diagnosed (Kruse et al., 2017a; Savchenko et al., 2017; Kruse et al., 2018). Moreover, traditional taxonomic classification based on spore size, color, and ornamentation, is tedious and requires a huge amount of spores (>50) of every species for comparing statistically (Inman et al., 2003). It is a very well known fact that accurate and rapid identification of phytopathogenic fungi is one of the most important prerequisites of disease monitoring and early warning, which provide solid foundation for the prevention and control of plant diseases (McCartney et al., 2003; Kumar et al., 2013; Fang and Ramasamy, 2015; Sharma et al., 2017; Kashyap et al., 2017; Kashyap et al., 2019; Chakdar et al., 2019). PCR based detection tests are more sensitive, reliable, and rapid compared to conventional morphological and phenotypic methods. Single locus DNA sequence studies were a common practice in fungi in the past; thus, numerous pieces of information have been accumulated in databases (Kruse et al., 2017a; Kruse et al., 2017b; Savchenko et al., 2017; Kruse et al., 2018). In particular, ribosomal DNA internal transcribed spacer (rDNA-ITS) sequences have been proven as a gold standard for the characterization of fungi (Schoch et al., 2012). The major merits of ITS include: short length (~500 bp), high accuracy, availability of universal primers, and strong amplification signal rates (Hillis and Dixon, 1991; Martin and Rygiewicz, 2005; Ji et al., 2017). Additionally, ITS1 has high copy numbers in the genome which gave high concentration of amplified fragments thus enabling the reliable detection from DNA of limited spores (<10) in PCR reaction (Tan and Murray, 2006). Thus, the rDNA-ITS region has been widely exploited to distinguish various smut and bunt diseases. PCR amplification using primers derived from the DNA sequence of the ITS region of ribosomal DNA is reported as an ideal marker for the detection of *Ustilago esculenta* in water oat tissue (Chen and Tzeng, 1999) and *Ustilago hordei* in leaves of susceptible and resistant barley varieties (Willits and Sherwood, 1999). Kochanová et al. (2004) simultaneously detected *T. controversa* and *T. tritici* through the primers made from the ITS1 region. Chen et al. (2016) reported rapid PCR based assay for the detection of rice kernel smut diseases by identifying specific pair of primers for the ITS1 region of *T. horrida*. Several studies have been reported for the identification and differentiation of smut and bunt fungi especially of *Tilletia* and *Ustilago* genus by using PCR with ITS derived species specific markers (Zhou et al., 2003; Tan and Murray, 2006; Kruse et al., 2017b). Till date, no molecular markers are available that could distinguish and diagnose *U. agropyri* from other related and unrelated fungal plant pathogens. Therefore, the study was undertaken to identify the molecular signature of *U. agropyri* for devising a simple and rapid detection protocol for *U. agropyri* in wheat plants and soils.

The present study is the first attempt to report molecular signature of *U. agropyri* and PCR based assay for the detection and identification of *U. agropyri* in the wheat plants and soil. Specifically, oligonucleotide primers were designed from the ITS region and were validated for the detection of fungi in plants as well as on the field soil.

## Materials and Methods

### Field Survey and Sampling

A detailed description of the fungal isolates used in the current study is given in **Table 1**. Fifty different isolates of *U. agropyri* were collected from 2015 to 2019 from various regions of North-Western India. Generally, an annual crop health field survey is conducted every year to assess the wheat crop situation in the country. During these surveys, one halts at random fields after a distance of about 35–40 km and monitors the crop health situation. During these surveys diseased samples with typical symptoms of wheat flag smut were collected (**Table 1**). Procedurally, for the collection of infected plant tissue, each diseased wheat leaf, sheath, and stem was clamped with sterile forceps, and then the black powdery teliospores were shacked off and collected in a butter paper bag. The *U. agropyri* teliospores of 10  mg were treated as a sample. During 2019, 11 different composite soil samples from the fields showing flag smut infection on wheat were also collected (**Table S1**). For soil sampling, five subsoil samples of about 50 g each were gathered from each infected field in a depth of 0–5 cm and mixed to obtain a composite sample. Each composite soil sample was sieved through a 40-mesh strainer and collected into a small sealed bag. These bags were properly labeled and stored at 4°C for further processing.

**Table 1:** Fungal isolates used in the present study to evaluate primer specificity in PCR assays.

S. No.	Fungus	Isolate Code	Diseases/host	State	Year of collection	Specificity obtained with primer pair	
						UA-17F/UA-519R	UA-15F/UA-562R
1.	*Urocystis agropyri*	FLS1	Flag smut/Wheat	Uttrakhand, India	2015	+	+
2.	*U. agropyri*	FLS2	Flag smut/Wheat	Uttar Pradesh, India	2015	+	+
3.	*U. agropyri*	FLS3	Flag smut/Wheat	Jammu, India	2015	+	+
4.	*U. agropyri*	FLS4	Flag smut/Wheat	Jammu, India	2015	+	+
5.	*U. agropyri*	FLS5	Flag smut/Wheat	Himachal Pradesh, India	2015	+	+
6.	*U. agropyri*	FLS6	Flag smut/Wheat	Punjab, India	2015	+	+
7.	*U. agropyri*	FLS7	Flag smut/Wheat	Uttar Pradesh, India	2015	+	+
8.	*U. agropyri*	FLS8	Flag smut/Wheat	Uttar Pradesh, India	2015	+	+
9.	*U. agropyri*	FLS9	Flag smut/Wheat	Jammu, India	2015	+	+
10.	*U. agropyri*	FLS10	Flag smut/Wheat	Himachal Pradesh, India	2015	+	+
11.	*U. agropyri*	FLS11	Flag smut/Wheat	Himachal Pradesh, India	2015	+	+
12.	*U. agropyri*	FLS12	Flag smut/Wheat	Punjab, India	2015	+	+
13.	*U. agropyri*	FLS13	Flag smut/Wheat	Haryana, India	2015	+	+
14.	*U. agropyri*	FLS14	Flag smut/Wheat	Himachal Pradesh, India	2016	+	+
15.	*U. agropyri*	FLS15	Flag smut/Wheat	Himachal Pradesh, India	2016	+	+
16.	*U. agropyri*	FLS16	Flag smut/Wheat	Uttrakhand, India	2016	+	+
17.	*U. agropyri*	FLS17	Flag smut/Wheat	Uttrakhand, India	2016	+	+
18.	*U. agropyri*	FLS18	Flag smut/Wheat	Haryana, India	2016	+	+
19.	*U. agropyri*	FLS19	Flag smut/Wheat	Rajasthan, India	2016	+	+
20.	*U. agropyri*	FLS20	Flag smut/Wheat	Rajasthan, India	2016	+	+
21.	*U. agropyri*	FLS21	Flag smut/Wheat	Rajasthan, India	2016	+	+
22.	*U. agropyri*	FLS22	Flag smut/Wheat	Rajasthan, India	2016	+	+
23.	*U. agropyri*	FLS23	Flag smut/Wheat	Himachal Pradesh, India	2016	+	+
24.	*U. agropyri*	FLS24	Flag smut/Wheat	Himachal Pradesh, India	2016	+	+
25.	*U. agropyri*	FLS25	Flag smut/Wheat	Himachal Pradesh, India	2016	+	+
26.	*U. agropyri*	FLS26	Flag smut/Wheat	Punjab, India	2016	+	+
27.	*U. agropyri*	FLS27	Flag smut/Wheat	Himachal Pradesh, India	2016	+	+
28.	*U. agropyri*	FLS28	Flag smut/Wheat	Himachal Pradesh, India	2016	+	+
29.	*U. agropyri*	FLS29	Flag smut/Wheat	Jammu, India	2016	+	+
30.	*U. agropyri*	FLS30	Flag smut/Wheat	Uttar Pradesh, India	2016	+	+
31.	*U. agropyri*	FLS31	Flag smut/Wheat	Uttrakhand, India	2016	+	+
32.	*U. agropyri*	FLS32	Flag smut/Wheat	Uttar Pradesh, India	2016	+	+
33.	*U. agropyri*	FLS33	Flag smut/Wheat	Uttar Pradesh, India	2016	+	+
34.	*U. agropyri*	FLS34	Flag smut/Wheat	Jammu, India	2016	+	+
35.	*U. agropyri*	FLS35	Flag smut/Wheat	Himachal Pradesh, India	2017	+	+
36.	*U. agropyri*	FLS36	Flag smut/Wheat	Himachal Pradesh, India	2017	+	+
37.	*U. agropyri*	FLS37	Flag smut/Wheat	Punjab, India	2017	+	+
38.	*U. agropyri*	FLS38	Flag smut/Wheat	Haryana, India	2017	+	+
39.	*U. agropyri*	FLS39	Flag smut/Wheat	Himachal Pradesh, India	2017	+	+
40.	*U. agropyri*	FLS40	Flag smut/Wheat	Himachal Pradesh, India	2017	+	+
41.	*U. agropyri*	FLS41	Flag smut/Wheat	Uttrakhand, India	2017	+	+
42.	*U. agropyri*	FLS42	Flag smut/Wheat	Uttrakhand, India	2017	+	+
43.	*U. agropyri*	FLS43	Flag smut/Wheat	Haryana, India	2017	+	+
44.	*U. agropyri*	FLS44	Flag smut/Wheat	Rajasthan, India	2017	+	+
45.	*U. agropyri*	FLS45	Flag smut/Wheat	Rajasthan, India	2017	+	+
46.	*U. agropyri*	FLS46	Flag smut/Wheat	Rajasthan, India	2018	+	+
47.	*U. agropyri*	FLS47	Flag smut/Wheat	Rajasthan, India	2018	+	+
48.	*U. agropyri*	FLS48	Flag smut/Wheat	Himachal Pradesh, India	2018	+	+
49.	*U. agropyri*	FLS49	Flag smut/Wheat	Himachal Pradesh, India	2018	+	+
50.	*U. agropyri*	FLS50	Flag smut/Wheat	Himachal Pradesh, India	2018	+	+
51.	*Tilletia indica*	PB-1	Karnal bunt/Wheat	Punjab, India	2019	−	−
52.	*T. indica*	HP-1	Karnal bunt/Wheat	Himachal Pradesh, India	2019	−	−
53.	*T. indica*	UP-1	Karnal bunt/Wheat	Uttar Pradesh, India	2019	−	−
54.	*T. indica*	HR-1	Karnal bunt/Wheat	Haryana, India	2019	−	−
55.	*Tilletia caries*	TC-1	Hill bunt/Wheat	Uttrakhand, India	2017	−	−
56.	*Ustilago hordei*	BUH-1	Covered smut/Barley	Haryana, India	2019	−	−
57.	*Ustilago tritici*	UT-1	Loose smut/Wheat	Haryana, India	2019	−	−
58.	*Ustilago nuda* f. sp. *hordei*	BUN-1	Loose smut/Barley	Haryana, India	2019	−	−
59.	*Fusarium graminareum*	FGA-1	Head Scab/Wheat	Assam, India	2019	−	−
60.	*F. graminareum*	FGP-1	Head Scab/Wheat	Punjab, India	2019	−	−
61.	*F. graminareum*	FGWB-1	Head Scab/Wheat	West Bengal, India	2020	−	−
62.	*Alternaria triticina*	WAT-1	Black point/wheat	Punjab, India	2019	−	−
63.	*Alternaria triticina*	WAT-2	Black point/wheat	Uttar Pradesh, India	2019	−	−
64.	*Bipolaris soronkiniana*	BS-1	Leaf blight/Wheat	Haryana, India	2018	−	−
65.	*Bipolaris soronkiniana*	BS-2	Leaf blight/Wheat	West Bengal, India	2018	−	−
66.	*Bipolaris soronkiniana*	BS-3	Leaf blight/Wheat	Uttar Pradesh, India	2018	−	−
67.	*Pyrenophora* *tritici-repentis*	PTR-1	Tan spot/Wheat	Karnataka, India	2018	−	−
68.	*Pyrenophora* *tritici-repentis*	PTR-2	Tan spot/Wheat	Himachal Pradesh, India	2018	−	−
69.	*Alternaria alternata*	AA	Leaf blight/Wheat	Haryana, India	2018	−	−
70.	*Sclerotium rolfsii*	SR-1	foot and root rot/wheat	Karnataka, India	2018	−	−
71.	*Rhizoctonia solani*	RRS-4	Sheath blight/rice	Haryana, India	2018	−	−

+significant amplification; −no significant amplification.

### Genomic DNA Extraction

Total genomic DNA of fungal isolates and plant tissues was extracted using the Cetyltrimethyl-ammonium bromide (CTAB) method described by Kumar et al. (2013). Ungerminated teliospores of *U. agropyri*, *Tilletia indica*, *Tilletia caries, Ustilago triti* ***c*** *i*, and *Ustilago nuda* f. sp. *hordei* collected from various regions were directly processed for genomic DNA isolation using the similar procedure of Kumar et al. (2013). The mycelia of fungal isolates (*Fusarium graminareum*, *Alternaria triticina*, *Bipolaris soronkiniana*, *Pyrenophora tritici-repentis*, *Alternaria alternata*, *Sclerotium rolfsii*, and *Rhizoctonia solani*) were raised on potato dextrose broth (Hi Media, India) for seven days at 25 ± 2°C. The mycelial mat of each isolate was separated through sterile Whatman filter paper and ground to fine powder with mortar and pestle by adding liquid nitrogen. Similarly, composite soil sample (10 g) was processed for total DNA isolation by employing PowerSoil^®^ DNA Isolation Kit (MoBio Laboratories, Carlsbad, CA). DNA was quantified by loading 1 μl of DNA in BioDrop Touch PC + Spectrophotometer (BioDrop, Cambridge shire, UK) and diluted to 50 ng μl^−1^ for further PCR based assays.

### Designing and Development of *U. agropyri* Specific Primers

**Figure 1:**
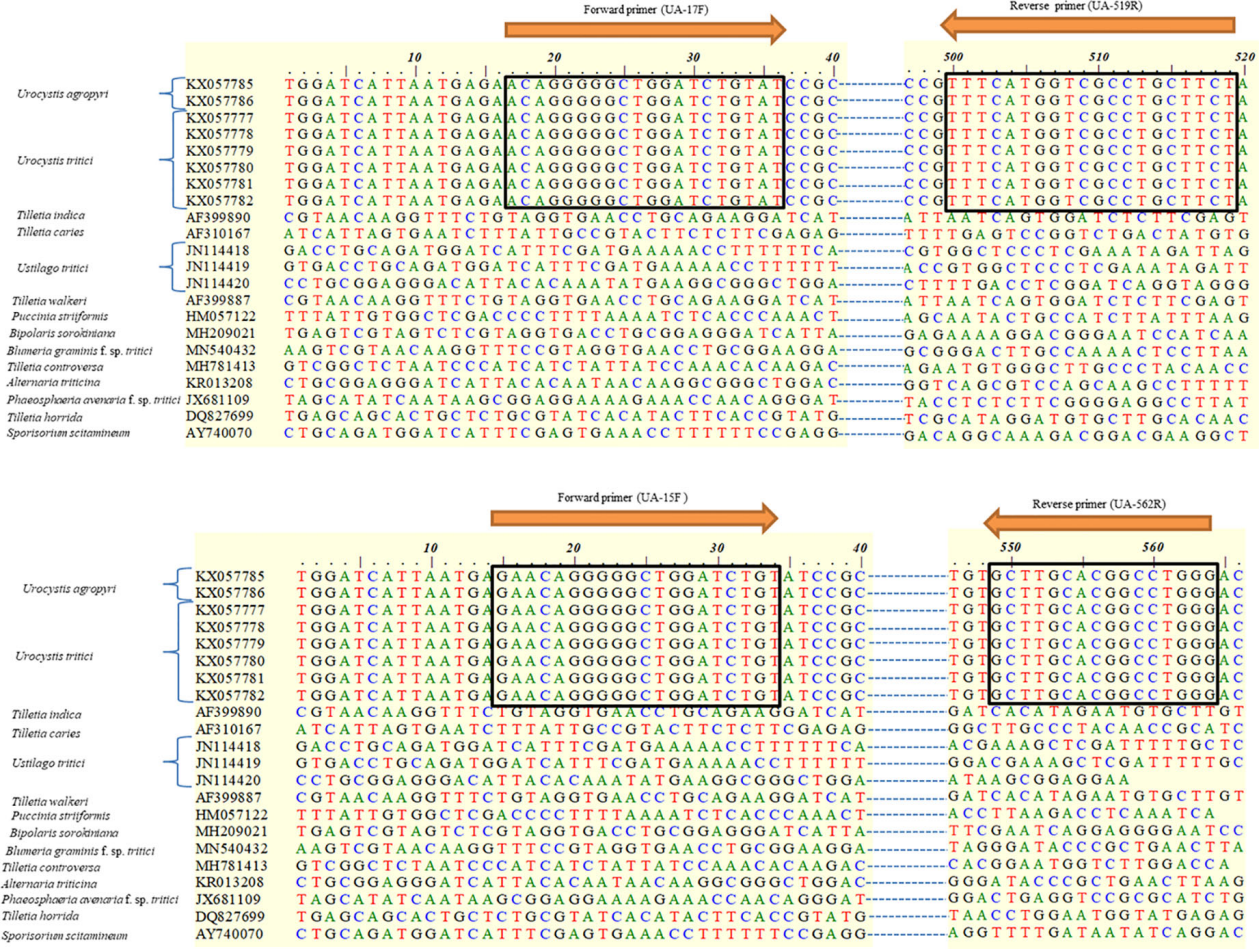
Alignment of consensus sequences of internal transcribed spacer (ITS) regions of the ribosomal DNA (rDNA) used for selecting diagnostic primers which are indicated in box. Numbers on the top refer to nucleotide positions of NCBI GenBank accession KX057786. The two arrows indicate the forward (UA-17F and UA-15F) and reverse (UA-519R and UA-562R) primers identified for the amplification of *U. agropyri* specific DNA. The specificity of the designed primer sets was indicated by making *in silico* comparison with the nonredundant (nr) sequences from other fungal pathogens (*Tilletia indica*, *T. caries*, *Ustilago tritici*, *T. walkeri*, *Puccinia striiformis*, *Bipolaris sorokiniana*, *Blumeria graminis* f. sp. *tritici*, *T. controversa*, *Alternaria triticina*, *Phaeosphaeria avenaria* f. sp. *tritici*, *T. horrida*, and *Sporisorium scitamineum*).

To design the species specific primer (**Table 2**) for PCR based detection of *U. agropyri*, ITS rDNA sequence of *U. agropyri* voucher WSP 72765 (Accession No. KX057786) was used. The sequences were analyzed for homology with other fungal pathogens including, *T. indica*, *T. caries*, *T. horrida*, *T. walkeri*, *T. controversa*, *Ustilago tritici*, *Puccinia striiformis*, *Bipolaris sorokiniana*, *Blumeria graminis* f. sp. *tritici*, *Alternaria triticina*, *Sporisorium scitamineum*, and *Phaeosphaeria avenaria* f. sp. *tritici* (**Figure 1**). Primers were designed from the regions conserved for *U. agropyri* (Syn = *Urocystis tritici*) and unmatched with other closely related species using Primer 3 program (Untergasser et al., 2012). OligoAnaylzer (https://www.idtdna.com/pages/tools/oligoanalyzer) program was used for checking oligonucleotide properties such as melting temperature, mismatches and formation of hairpins and dimers *etc.* The specificity of the designed primer sets were also checked *in silico* by searching against the nonredundant (nr) sequences from other organisms *via* Primer- Basic Local Alignment Search (BLAST) tool (https://www.ncbi.nlm.nih.gov/tools/primer-blast) of National Center for Biotechnology Information (NCBI) with default parameters. The primers were compared to the database by using FASTA and BLAST to confirm specificity. The PCR conditions were optimized using *U. agropyri* FLS1 as template DNA. PCR reaction cocktail (25 μl) was prepared by incorporating 1 μl of template DNA (50 ng μl^−1^), 12.5 μl of Go Taq Green master mix (Promega Biotech India Pvt. Ltd), 1 μl of each primer (10 μM) (**Table 2**), and total volume (25 μl) was adjusted by double distilled water. PCR reaction without DNA template was served as negative control (NC). The thermal cycler (Q cycler 96, Hain Life Science, UK) was programmed as initial denaturation at 94°C for 5 min than 35 cycles of 1 min at 94°C, 45 s at 54°C and 30 s at 72°C and final extension at 72°C for 5 min, and holding at 4°C. Without DNA template served as negative control. The amplification results were visualized through electrophoresis using 1% agarose gel in 1× TAE buffer for 45 min at 90 V with 100 bp DNA ladder (Bangalore Genei, India).

**Table 2:** Primers developed in the present study.

Primer	Sequence (5′-3′)	Amplicon size (bp)	T_a_(^°^C)
UA-17F	ACAGGGGGCTGGATCTGTAT	503	54
UA-519R	AGAAGCAGGCGACCATGAAA		
UA-15F	GAACAGGGGGCTGGATCTGT	548	54
UA-562R	CCCAGGCCGTGCAAGC		

### Specificity and Sensitivity Assay

The specificity of the primers to *U. agropyri* was determined by performing PCR with different concentrations of DNA and teliospores of *U. agropyri* using the aforementioned standardized PCR procedure.

The PCR assay to detect *U. agropyri* FLS1 was determined by employing two different protocols. In the first method, sensitivity of the PCR assay was evaluated by detecting serially diluted DNA concentrations of *U. agropyri* FLS1 from 10 ng μl^−1^ to 0.01 pg μl^−1^ as a template. PCR reactions were performed as previously described with *U. agropyri* specific UA-17F/UA-519R and UA-15F/UA-562R primer sets (**Table 2**). The experiment was performed twice.

In the second protocol, a calibration towards the sensitivity of the developed PCR protocol was executed by serial dilutions with standard known concentration of *U. agropyri.* FLS1 teliospores (1 mg of teliospore/1 g of soil) were added to sterile soil containing 4.2 × 10^4^ teliospores. The desired teliospore concentration was adjusted by spore counting in a hemocytometer. Ten-fold dilutions were prepared by diluting the teliospore suspension to get the final concentration up to 0.42 teliospore spores g^−1^ soil, and DNA isolation was performed as per the procedure mentioned earlier. The PCR assay was conducted by using the individual spore suspensions along with a control (NC), substituting the template DNA with sterile water only. The PCR master mixture, thermal amplification profile, and electrophoresis conditions were the same as described earlier. This assay was performed twice.

### Detection of *U. agropyri* in Wheat Plants

Polymerase chain reaction (PCR) mediated detection of *U. agropyri* in wheat tissues of six wheat cultivars (*viz*., PBW343, PBW550, HS673, WH1105, DBW107, and HI1563) grown in flag smut sick plot at two different time intervals (20 and 40 days after sowing) was performed. DNA was extracted from leaves and stems of six infected wheat varieties (**Figure 3**). Similarly, as a negative control (NC), wheat leaf and stem samples of these varieties were collected from wheat fields with no previous history of occurrence of flag smut disease. About 1 g of fresh tissue from infected and healthy wheat plants was processed according to the method described previously. PCR reaction was performed using the aforementioned optimized PCR conditions. DNA of *U. agropyri* FLS1 was used as a positive control.

### Detection of *U. agropyri* in Soil Samples

To test the potential use of the developed primer set (UA-17F/UA-519R and UA-15F/UA-562R) (**Table 2**) for detecting the presence of *U. agropyri* in soil, two independent experiments were executed.

In the first experiment, random plots within a field with and without previous flag smut disease history at wheat pathological experimental farm, ICAR-IIWBR, Karnal, India were selected. Soil samples were taken at a depth of 0–5 cm. Three randomly selected points from each plot with flag smut history were combined into a single pooled sample. Similarly, field soil plots without previous flag smut disease history were treated as a control plot. The sampled soil was later checked and confirmed by comparing spore morphology with characteristics microscopic features of *U. agropyri* under microscope. Each pooled soil sample was air-dried, sieved and thoroughly mixed before the assay and strained through a sieve (40-mesh size). The resultant fine soil was collected into a small sealed bag. These bags were properly labeled and stored at 4°C for further processing. Total DNA was extracted from soil samples (10 g) with PowerSoil^®^ DNA isolation Kit (MoBio Laboratories, Carlsbad, CA). The quality and concentration of the DNA was checked by loading 1 μl of DNA in BioDrop Touch PC + Spectrophotometer (BioDrop, Cambridge shire, UK). PCR reaction was performed using the aforementioned optimized PCR conditions. All the PCR reactions were repeated twice and always run with a positive control (PC) representing a known template DNA of *U. agropyri* FLS1 (PC1) and two negative control (NC) *i.e.* NC1 (without DNA template) and NC2 (DNA extracted from healthy soil free from *U. agropyri* spores).

In the second experiment, PCR amplification for the detection of *U. agropyri* in the soil with *U. agropyri*-specific primer sets (UA-17F/UA-519R and UA-15F/UA-562R) was performed by using the genomic DNA of 11 different composite soil samples representing natural field soil of different localities (**Table S1**). The collection of soil samples, soil DNA isolation, PCR reaction, and gel electrophoresis was performed using the aforementioned procedures. DNA of *U. agropyri* FLS1 was used as a positive control (PC).

## Results

### Development and Validation of Species Specific *U. agropyri* Primers

Based on the alignment of *Urocystis agropyri* ITS rDNA sequence of *U. agropyri* voucher WSP 72765 (Accession No. KX057786) with the sequences of other pathogens of wheat submitted to the NCBI database by nucleotide BLAST analysis and Clustal W (bioedit), the two sets of specific primers have been designed (**Table 2**). These two sets of forward and reverse primer pairs (UA-17F/UA-519R and UA-15F/UA-562R) lie within the ITS1-5.8S rDNA-ITS2-28S rDNA region of *U. agropyri* as shown in **Figure 1**.

**Figure 2:**
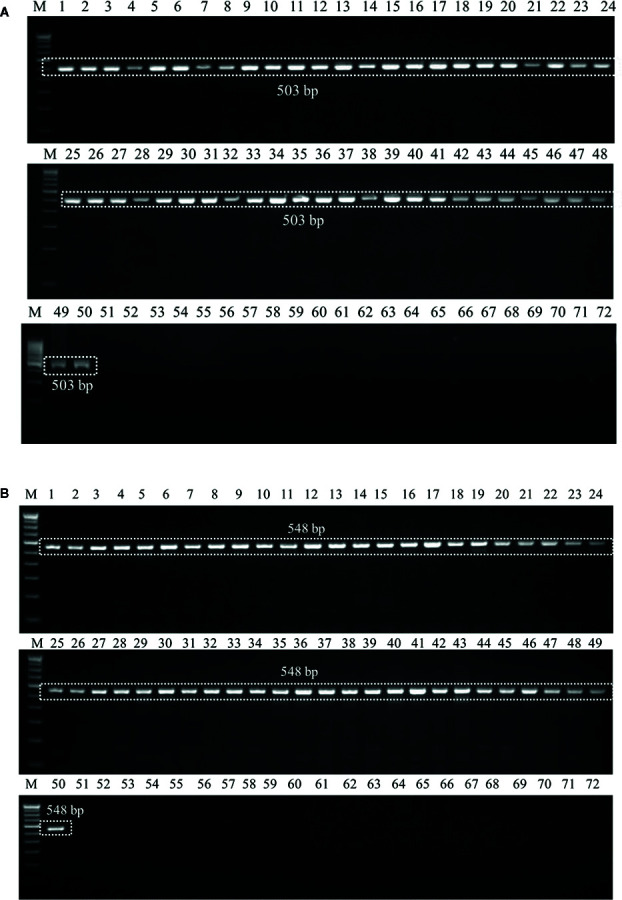
**(A)** Agarose gel electrophoresis showing the PCR product (503 bp) amplified using the UA-17F/UA-519R primer sets from the fungal isolates listed in **Table 1**. M: 100 bp DNA ladder; Lane 72 indicates negative control (NC) without DNA template. **(B)** Agarose gel electrophoresis showing the PCR product (548 bp) amplified using the UA-15F/UA-562R primer sets from the fungal isolates listed according to **Table 1**. M: 100 bp DNA ladder; Lane 72: Negative control (NC) without DNA template.

The ability of the primer pair UA-17F/UA5-19R and UA-15F/UA-562R (**Table 2**) was first evaluated by using the genomic DNA of 50 geographical distinct isolates of *U. agropyri* and 21 isolates of related and unrelated fungal pathogens (**Table 1**) as template for PCR assay. PCR based amplicon generation (**Figures 2A, B** **)** revealed that each set of the designed primers produced only a single band of 503 and 548 bp from the DNA of *U. agropyri* but not from the other 21 tested fungal pathogens, nullifying the events of cross species amplification for the developed PCR assay for *U. agropyri* detection.

### Detection and Confirmation of *U. agropyri* in Wheat Plants

**Figure 3:**
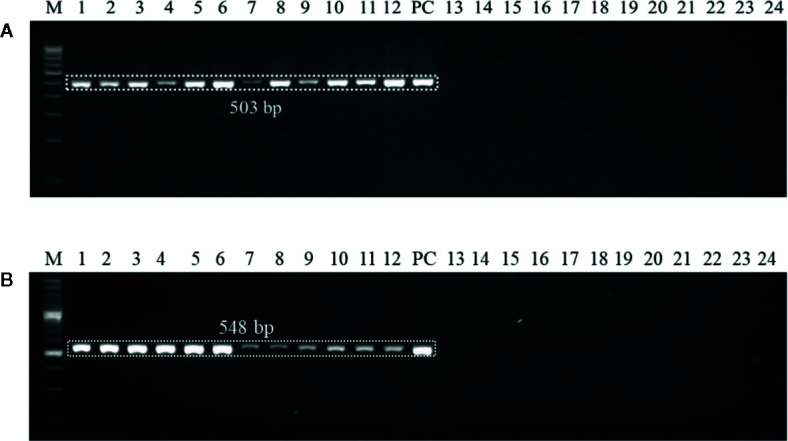
Polymerase chain reaction (PCR) mediated detection of *U. agropyri* in wheat tissues of six wheat cultivars (*viz*., PBW343, PBW550, HS673, WH1105, DBW107, and HI1563) using UA-17F/UA-519R **(A)** and UA-15F/UA-562R **(B)** primer sets. Lanes 1–12: Wheat tissue (*viz*., PBW343, PBW550, HS673, WH1105, DBW107, and HI1563) sampled from *U. agropyri* disease sick plot at 20 DAS (Lanes 1–6) and 40 DAS (7–12); Lanes 14–24: Negative control and include wheat tissue (*viz*., PBW343, PBW550, HS673, WH1105, DBW107, and HI1563) sampled from sterilized healthy soil free *U. agropyri* spores at 20 DAS (Lanes 13–18) and 40 DAS (Lanes 19–24); M, 100 bp DNA ladder; PC, Positive control of *U. agropyri* FLS1 DNA only.

Genomic DNAs extracted from the six wheat cultivars (*viz*., PBW343, PBW550, HS673, WH1105, DBW107, and HI1563) sown under *U. agropyri* sick soil and sterile soil without *U. agropyri* were used as templates to verify whether the optimized polymerase chain reaction assay can detect *U. agropyri* in the plant materials. Following PCR amplification with UA-17F/UA-519R, a single 503 bp product was generated from plant DNA extracted from the all the six cultivars grown under the disease sick plot along with the positive control of *U. agropyri* FLS1 DNA, indicating that the primer pair can detect *U. agropyri* at both the sampling points *i.e.* 20 and 40 DAS (**Figure 3A**). Similarly, an amplicon of 548 bp was produced from a plant DNA extracted from all the six test cultivars collected from the disease sick plot at both the sampling times (20 and 40 DAS) along with the positive control of *U. agropyri* FLS1 DNA when UA-15F/UA-562R primer pairs was used (**Figure 3B**). However, no amplification was observed using UA-17F/UA-519R and UA-15F/UA-562R primers on the genomic DNAs extracted from six varieties sampled at two different time points from healthy soil free from *U. agropyri* spores (**Figures 3A, B**).

### PCR Sensitivity Assay

**Figure 4:**
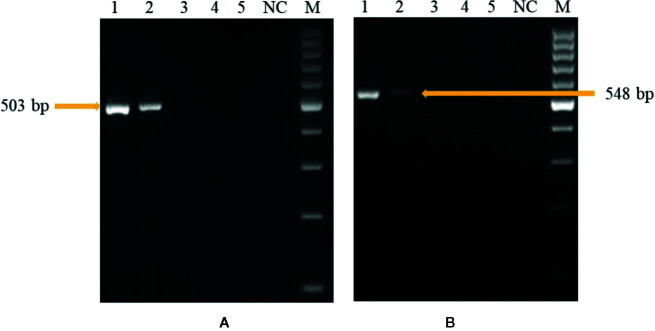
Polymerase chain reaction (PCR) based sensitivity test of the UA-17F/UA-519R **(A)** and UA-15F/UA-562R **(B)** marker with different amounts of DNA template of *U. agropyri* FLS1. M: Marker; Lanes 1–5 indicate 10 ng, 1 ng, 100 pg, 10 pg, and 0.1 pg *U. agropyri* FLS1 DNA, respectively; NC: Negative control without DNA template.

In the first experiment, sensitivity level of the developed PCR assay was monitored by using purified DNA of *U. agropyri* FLS1 (100 ng, 10 ng, 1 ng, 0.1 g, 0.01 ng, 1 pg, and 0.1 pg, respectively) as a genomic DNA template. The research findings of the study revealed that the developed PCR assay could detect at least 1 ng genomic DNA of *U. agropyri* when UA-17F/UA-519R (**Figure 4A**) and UA-15F/UA-562R (**Figure 4B**) primer sets were used in the PCR assay. Following amplifications, two amplicons of 503 bp (using primer UA-17F/UA-519R) and 548bp (using primer UA-15F/UA-562R) sizes were observed in gels in the PCR reactions containing up to 1 ng DNA of *U. agropyri* (**Figure 4**).

**Figure 5:**
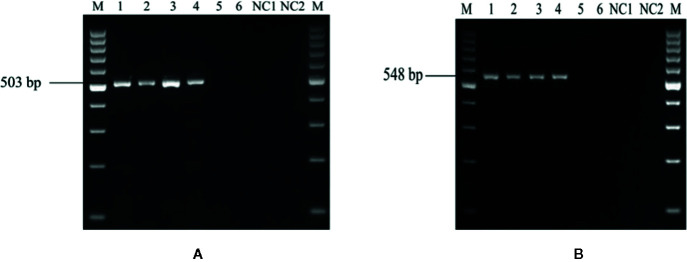
Polymerase chain reaction (PCR) based sensitivity test of the UA-17F/UA-519R **(A)** and UA-15F/UA-562R **(B)** marker with different amounts of DNA template of *U. agropyri* FLS1 teliospore suspension (4.2 × 10^4^ to 4.2 × 10^−1^ spores g^−1^ soil) mixed with sterile soil. M: Marker; Lanes 1–6 indicate serial dilutions (1:10) of the fungal spores’ suspension (*i.e.* Lane 1: 42,000 spores g^−1^ soil, Lane 2: 4,200 spores g^−1^soil, Lane 3: 420 spores g^−1^ soil, Lane 4: 42 spores g^−1^ soil, Lane 5: 4.2 spores g^−1^ soil, Lane 6: 0.42 spores g^−1^ soil, respectively). NC1, Negative control employing DNA of sterilized healthy soil without *U. agropyri* spores; NC2, Negative control without DNA template.

In the second experiment, for the identification of the detection limit of the PCR assay, different amounts of DNA extracted from soil mixed with *U. agropyri* teliospore suspension (4.2 × 10^4^ to 4.2 × 10^−1^ spores g^−1^soil) with sterile soil used as PCR start material. Electrophoresis of the amplified fragments revealed that both the primers UA-17F/UA-519R (**Figure 5A**) and UA-15F/UA-562R (**Figure 5B**) produced single band of 503 and 548 bp, respectively, in the PCR aliquots containing up to 42 spores g^−1^ soil (**Figure 5**). No amplification with any of the primer pair was observed when DNA of ≤42 spores g^−1^ soil was used in the reaction mixture. Moreover, the experiments replicated twice and revealed no significant variation in the results.

### Detection of *U. agropyri* in Soil

**Figure 6:**
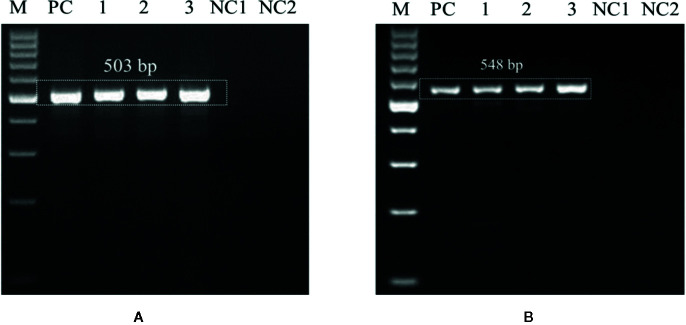
Agarose gel electrophoresis displaying the PCR products amplified by UA-15F/UA-562R **(A)** and UA-17F/UA-519R **(B)** primer sets using field soil DNA derived from *U. agropyri* infested disease sick plot and sterilized healthy soil without *U. agropyri*. M, 100 bp DNA marker; PC, PC: Positive control of *U. agropyri* FLS1 DNA only; NC1, Negative Control where addition of sterile water without DNA template of soil of disease sick plot; NC2, Negative control employing DNA of healthy soil without *U. agropyri* spores.

**Figure 7:**
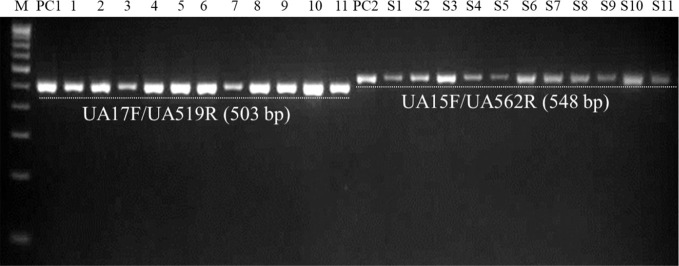
PCR-mediated detection of *U. agropyri* in field soil samples collected from different locations using UA-17F/UA-519R (Lanes 1–11) and UA-15F/UA-562R (Lanes S1–S11) primer sets. M, 100 bp DNA ladder; PC1, Positive control of *U. agropyri* FLS1 DNA for UA-17F/UA-519R primer set; PC2, Positive control of *U. agropyri* FLS1 DNA for UA-15F/UA-562R primer set.

The optimized PCR assay was performed to check *U. agropyri* presence by executing two different experiments (**Figures 6** and **7**). The results of agarose gel electrophoresis of the first experiment (**Figure 6**) displayed the PCR products amplified by UA-17F/UA-519R and UA-15F/UA-562R primers sets with specific single band of 503 bp and 548 bp, respectively, in all the soil samples derived from the *U. agropyri* infested disease sick plot (**Figure 6**). No amplification was visualized in the soil sample taken from the healthy plot with no prior history of wheat flag smut disease (**Figure 6**).

Similarly, agarose gel electrophoresis results of another independent testing of field soil samples demonstrated that both the primer pairs (UA-17F/UA-519R and UA-15F/UA-562R) resulted in the amplification of single and clear band of 503 bp and 548 bp, respectively, in the genomic DNA extracted from all the eleven soil samples collected from the flag smut infested fields from different geographical regions (**Figure 7**).

## Discussion

The current research describes a sensitive and rapid molecular procedure to identify and detect *U. agropyri* in wheat plants and soil. Numerous genetic markers have been developed to identify and detect smut and bunt fungi attacking various cereal crops (Josefsen and Christiansen, 2002; Tan et al., 2009; Gao et al., 2010; Kashyap et al., 2011; Wunderle et al., 2012; Chen et al., 2016); however, to the best of our knowledge, the current study presents the first report on the development of novel species-specific markers and their application to detect the presence of *U. agropyri* infection in wheat plant and inoculum in the soil.

Accurate and speedy detection process for *U. agropyri* identification in wheat crop is decisive for the effective implementation of regulatory procedures linked with surveillance and quarantine in the wheat trade at the global level. Detection assays employing PCR procedures have been developed and optimized for different types of plant pathogens, including viruses, bacteria, and fungi (Kumar et al., 2013; Singh et al., 2014; Kashyap et al., 2016; Sharma et al., 2017; Chakdar et al., 2019; Kaur et al., 2020). These PCR based assays found special attentions in various diagnostic laboratories due to manifold merits. For instance, these assays are extremely sensitive and highly specific; require minute quantity of plant tissue; and commercial kits exist for quality DNA isolation from any kind of fungal pathogen, plant, and soil samples. Most strikingly, PCR based techniques are reasonably easy to set up and execute, and reports can be generated swiftly, generally possible within 24 h. In the present research, specific primer sets were designed for the rapid detection and identification of *U. agropyri* in wheat seedlings by computational analysis of the ITS region of rDNA of various fungi such as *U. agropyri*, *T. indica*, *T. foetida*, *U. tritici*, *U. hordei*, *F. graminarium*, *Bipolaris sorokiniana*, *A. alternata*, and *S. rolfsii*, *etc.* The ITS region is characterized by a long tandem DNA repeat available between ITS1 and ITS2 rRNA genes in the rDNA unit in the eukaryotes (Rai et al., 2016) owing to the fact this region epitomizes a high degree of variation between the closely related species and therefore, widely exploited for the studies related to molecular phylogeny and fungal taxonomy (Schoch et al., 2012). Several studies clearly pointed that the ITS region has the highest probability of successful identification for the wide range of fungi (Schoch et al., 2012; Kashyap et al., 2017). Therefore, to distinguish *U. agropyri* from other smut and bunt fungi (*T. indica*, *T. caries*, *U. tritici*, and *U. hordei*), the PCR method was devised by designing specific pairs of primers to generate single and specific bands of 503 bp and 548 bp of *U. agropyri* ITS1 + 5.8S +  ITS2 rDNA region. The primers designed in the current study did not reflect specific and desired amplicon generation from the genomic DNA of all the tested fungi, except *U. agropyri*, highlighting their precise and specific nature. A number of researchers have also described the usefulness of ITS barcodes or signature sequences to identify various seeds and soil borne plant pathogenic fungi as *T. tritici* (Josefsen and Christiansen, 2002), *T. horrida* (Chen et al., 2016), *T. indica* (Tan et al., 2009), *U. esculenta* (Chen and Tzeng, 1999), and *U. hordei* (Willits and Sherwood, 1999).

The PCR based technique developed for the detection and differentiation of *U. agropyri* from other smuts and bunts in present study has several advantages over earlier reported morphological and microscopic methodologies. The developed method relies on simple and basic techniques of biological sciences and utilizes basic chemicals for running PCR and small apparatus like electrophoresis unit and PCR machine which are relatively low price, economical to maintain, and simple to use. In the current developed method, direct use of teliospores for the diagnosis of *U. agropyri* has been reported, which in turn eliminates the requirement of unwieldy and time-taking culturing procedures (Savchenko et al., 2017). Moreover, the results of sensitivity experiments revealed that the present method requires very minute quantity of fungal mass (either only ~42 spores g^−1^ soil or 1 ng of genomic DNA of *U. agropyri*). The results of the sensitivity test are in conformity with Chen et al. (2016), who reported detection limit of conventional PCR up to the level of 25 spores of *T. horrida*/100 g rice seeds mixture or ≥100 pg genomic DNA of *T. horrida*. The conventional morphology based identification protocols usually require extensive knowledge and sufficient amount of spore count (~50 spores) for a particular species prediction (Inman et al., 2003; Tan et al., 2009; Chen et al., 2016). The major merit of the developed PCR assay can be very valuable in the quarantine studies, where time and accuracy of identification is of utmost significance. For instance, in the present study, in a 25 μl PCR reaction cocktail, the sensitivity limit was found as low as 1 ng of pure genomic DNA of *U. agroyri* by both the primer sets. Most noteworthy is the fact that the *U. agroyri* specific primer sets have the potential to amplify only *U. agroyri* from fungal structures, either in the soil or in different plant tissues at the initial stages of infection (20 DAS) when pathogen colonization and symptom expression initiate, providing clues regarding the robust sensitivity and accuracy of the developed assay. All these facts assist in the rapid disease diagnosis since there is no need to isolate and culture the fungus in order to identify it. Moreover, the detection is possible within a day; therefore, the developed approach can be utilized for screening several wheat cultivars and plant tissues in a short time span.

Various studies demonstrating the specificity of primers within bulk soil have been documented by several workers (Lievens et al., 2006; Qu et al., 2006; Canfora et al., 2016). However, it is worth to notice here that we are also reporting the application of designed primers (UA-17F/UA-519R and UA-15F/UA-562R) not in artificial soil conditions but also in farmers’ field under natural disease infection conditions, where multifarious microbial diversity exists, provides a complex milieu to trace particular fungal species. To the best of our knowledge, this study presents the first report of a PCR based technique for the detection and traceability of *U. agropyri* inocula in natural field soil.

In conclusion, the PCR assay developed in the current study is sensitive, brisk, versatile, and consistent. Therefore, this assay will be very useful for the study of pathogen biology, ecology, host–pathogen interactions and get immense perspective for addressing fundamental problems regarding flag smut of wheat. More importantly, this assay will be extremely practical for the detection of contamination of wheat with smut teliospores.

## Data Availability Statement

The datasets presented in this study can be found in online repositories. The names of the repository/repositories and accession number(s) can be found in the article.

## Author Contributions

PK and SK conceived and designed the work and drafted the manuscript. PK, SK, and PJ performed the sampling survey. PK and AS were involved in *in-silico* designing of the primers. PK, SK, and RK executed laboratory and field experiments. PJ and SK performed the data analysis. SK, DS and GS performed the final editing and proofing of the manuscript. All authors contributed to the article and approved the submitted version.

## Conflict of Interest

The authors declare that the research was conducted in the absence of any commercial or financial relationships that could be construed as a potential conflict of interest.
