# Supplementary material for: Molecular Diagnostic Assay for Rapid Detection of Flag Smut Fungus (Urocystis agropyri) in Wheat Plants and Field Soil
Source: Front Plant Sci. 2020 Jul 10;11:1039. doi: 10.3389/fpls.2020.01039 (PMC7366794; doi:10.3389/fpls.2020.01039)
Supplement: Supplementary file 1 [file Table_1.docx]

| Table S1  Geographical location, source and year of collection of soil samples from wheat fields | | | | | |
| --- | --- | --- | --- | --- | --- |
| **S. No.** | **Crop** | **Collection site(s)** |  | **Flag smut symptoms presence on plants parts** | **Year of soil sampling** |
| 1 | Wheat | Bibi Wala, Punjab |  | Leaf | 2019 |
| 2 | Wheat | Kutlupur, Punjab |  | Leaf , stem | 2019 |
| 3 | Wheat | Durgapura, Rajasthan |  | Leaf | 2019 |
| 4 | Wheat | Bikaner, Rajasthan |  | Leaf | 2019 |
| 5 | Wheat | UAS Nagar, Uttrakhand |  | Leaf | 2019 |
| 6 | Wheat | Pattharchatta, Uttrakhand |  | Leaf, stem | 2019 |
| 7 | Wheat | Una, Himachal Pradesh |  | Leaf, stem | 2019 |
| 8 | Wheat | Kangra, Himachal Pradesh |  | Leaf, stem | 2019 |
| 9 | Wheat | Karnal, Haryana |  | Leaf, stem | 2019 |
| 10 | Wheat | Gaddou Pur, Uttar Pradesh |  | Leaf, stem | 2019 |
| 11 | Wheat | Saraiya, Uttar Pradesh |  | Leaf | 2019 |
